# Automatic Generation of Accurate and Cost-Efficient Auxiliary Basis Sets

**DOI:** 10.1021/acs.jctc.3c00670

**Published:** 2023-09-04

**Authors:** Susi Lehtola

**Affiliations:** † Molecular Sciences Software Institute, Blacksburg, Virginia 24061, United States; ‡ Department of Chemistry, University of Helsinki, P.O. Box 55, FI-00014 Helsinki, Finland

## Abstract

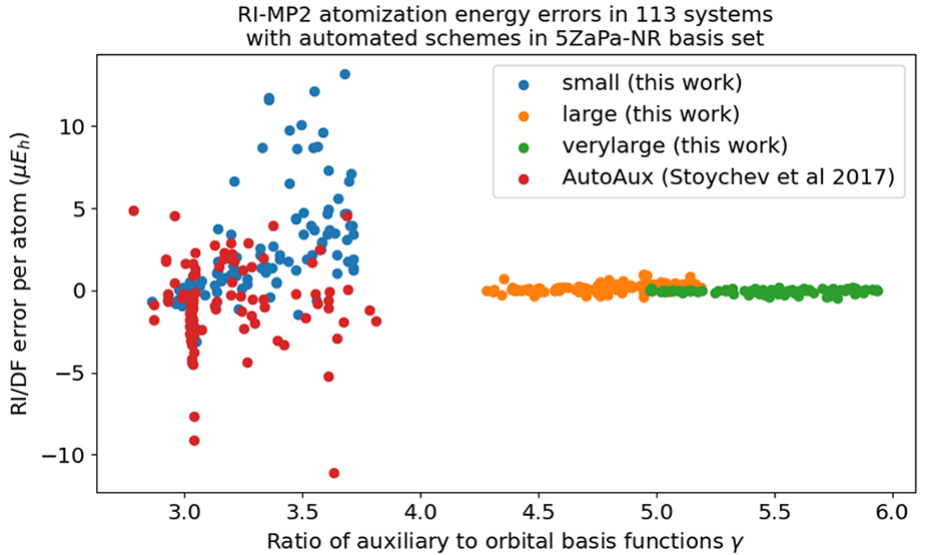

We have recently discussed an algorithm to automatically generate auxiliary basis sets (ABSs) of the standard form for density fitting (DF) or resolution-of-the-identity (RI) calculations in a given atomic orbital basis set (OBS) of any form, such as Gaussian-type orbitals, Slater-type orbitals, or numerical atomic orbitals [*J. Chem. Theory Comput.2021*, *17*, 6886]. In this work, we study two ways to reduce the cost of such automatically generated ABSs without sacrificing their accuracy. We contract the ABS with a singular value decomposition proposed by Kállay [*J. Chem. Phys.***2014**, *141*, 244113], used here in a somewhat different setting. We also drop the high-angular momentum functions from the ABS, as they are unnecessary for global fitting methods. Studying the effect of these two types of truncations on Hartree–Fock (HF) and second-order Møller–Plesset perturbation theory (MP2) calculations on a chemically diverse set of first- and second-row molecules within the RI/DF approach, we show that accurate total and atomization energies can be achieved by a combination of the two approaches with significant reductions in the size of the ABS. While the original approach yields ABSs whose number of functions *N*_bf_^ABS^ scales with the number of functions in the OBS, *N*_OBS_^bf^, as *N*_ABS_^bf^ = *γN*_OBS_^bf^ with the prefactor , the reduction schemes of this work afford results of essentially the same quality as the original unpruned and uncontracted ABS with γ ≈ 5–6, while an accuracy that may suffice for routine applications is achievable with a further reduced ABS with γ ≈ 3–4. The observed errors are similar at HF and MP2 levels of theory, suggesting that the generated ABSs are highly transferable and can also be applied to model challenging properties with high-level methods.

## 1 Introduction

The density fitting^1−5^ (DF) also known as the resolution of the identity^6^ (RI) technique has become one of the mainstays of quantum chemistry. The DF/RI approach is widely available in many program packages as an optional technique to accelerate self-consistent field (SCF) as well as post-Hartree–Fock calculations. It is used by default in some packages, such as Psi4(7) and Orca,^8^ while other packages like BAGEL^9^ and deMon2k(10) go even further by relying solely on the use of DF/RI for all calculations, thus foregoing traditional routines based on the exact electron repulsion integrals (ERIs).

Assuming that the basis-functions are real-valued, these ERIs are typically written in the Mulliken notation as
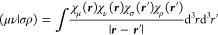
1where μ, ν, σ, and ρ are indices of atomic-orbital basis functions χ_μ_ that together form the orbital basis set (OBS). In the DF/RI approach, the two-electron integrals of the OBS are approximated with the help of an auxiliary basis set (ABS) as^6^
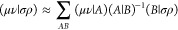
2where *A* and *B* are functions in the ABS, and (*A*|*B*)^−1^ denotes the *A*,*B* element of the inverse of the two-index Coulomb overlap matrix (*A*|*B*). Traditionally, ABSs are manually optimized for ground-state energy calculations for each OBS for each level of theory,^11,12^ but some automatic algorithms for generating an ABS from the given OBS have also been suggested;^13−18^ see ref (12) for a recent review.

While some of these automated schemes inherently assume the use of Gaussian basis sets,^14,15,17^ we recently suggested an automated algorithm which can be used with any type of atomic-orbital basis set. In short, the central idea of this scheme is to generate the ABS by choosing it such that it spans all one-center products χ_μ_χ_ν_ in the OBS to a given degree of precision.

Because the angular part is closed—the product of two spherical harmonics yielding a linear combination of spherical harmonics—the task can be reduced to studying radial functions that should be well-described by the sought-for ABS. As the one-center OBS products χ_μ_χ_ν_ can couple to angular momentum |*l*_μ_ – *l*_ν_| ≤ *L* ≤ *l*_μ_ + *l*_ν_, where *l*_μ_ and *l*_ν_ are the angular momenta of the functions χ_μ_ and χ_ν_, respectively, the algorithm of ref (18) works by assembling a list of normalized product radial functions
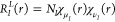
3for all possible angular momenta *L* in the auxiliary basis set, *N*_*I*_ being the normalization coefficient in eq 3. These product functions *R*_*I*_^*L*^ serve as candidate auxiliary basis functions for each shell *L*. The set of candidate radial functions can be prescreened by a pivoted Cholesky decomposition of the ERI tensor;^19^ this yields a more compact auxiliary basis set at no loss in accuracy.^18^

Next, the algorithm chooses the set of auxiliary radial functions for each angular momentum *L*, *R*_*I*_^*L*^(*r*), by a pivoted Cholesky decomposition of the one-center Coulomb overlap matrix (*R*_*I*_^*L*^|*R*_*J*_^*L*^), motivated by earlier work of Aquilante et al.^15^ The decomposition is carried out to the given threshold τ. The resulting set of functions is capable of reproducing all the functions in the candidates pool {*R*_*I*_^*L*^} to within the used threshold τ, thus affording an optimal^20^ black-box method to generate auxiliary basis sets.

The algorithm of ref (18) is applicable to any atomic-orbital basis sets: the necessary equations to compute the one-center Coulomb overlap matrix for Gaussian-type orbitals (GTOs) as well as Slater-type orbitals (STOs) are given in ref (18), while the necessary integrals for numerical atomic orbitals^21^ (NAOs) can easily be computed by quadrature.^22^

As a side note, we have also successfully used an analogous technique to cure OBS overcompleteness in SCF calculations by solving the Roothaan equations^23^***FC*** = ***SCE***, where ***F*** is the Fock matrix, ***S*** is the overlap matrix, and ***C*** and ***E*** are the matrix of orbital expansion coefficients and the corresponding diagonal matrix of orbital energies, in a subbasis chosen by the pivoted Cholesky decomposition of ***S***.^24,25^ Choosing the basis functions by a pivoted Cholesky decomposition of the (Coulomb) overlap matrix—which is a so-called Gram matrix—is analogous to using an optimal Gram–Schmidt orthogonalization.^12^

Even if automatically generated ABSs tend to be large compared with their possible manually optimized counterparts, they can be extremely useful for practical applications. Although an automatically generated auxiliary basis set may be several times larger than a manually optimized conterpart, the cost of DF/RI methods scales only linearly with the size of the ABS. The automatic auxiliary basis set machinery enables the use of the DF/RI technique in all OBSs, offering significant speedups in calculations compared to the use of the exact TEIs, because the factorization of eq 2 can be employed to formulate efficient implementations of many quantum chemical models. Therefore, DF/RI calculations with automatically generated basis sets tend to be not only considerably faster than calculations with exact ERIs, but also not that much slower than DF/RI calculations employing hand-optimized ABSs, provided that such ABSs exist for the employed OBS and the studied property at the considered level of theory.

Our previous study^18^ focused on showing that negligible errors in Hartree–Fock (HF) and second-order Møller–Plesset perturbation theory (MP2) total energies could be achieved with the technique proposed therein. The calculations in ref (18) were carried out with GTO basis sets to allow the straightforward computation of exact values without the DF/RI approximation.

In this work, we focus on improving the cost efficiency of the automatically generated ABS. We accomplish this along two routes. First, we consider contracting the ABS employing a singular value decomposition (SVD). The use of this technique to reduce the cost of RI/DF methods was originally suggested by Kállay^26^ within a different context: system-specific truncations of fixed ABSs in polyatomic calculations. In this work, the contraction of the ABS serves to restore the energetic connection of the autogenerated basis to the two-electron integrals, which was severed in the algorithm of ref (18) that was laid out above. The contraction procedure is especially useful in the case of contracted Gaussian OBSs, as the resulting contracted ABSs turn out to have fewer auxiliary basis functions. The contraction procedure is also seen to be beneficial with uncontracted Gaussian basis sets as it results in a reduced number of auxiliary basis functions. We expect the contraction procedure to be useful especially in the case of NAOs, because the recontraction of NAOs can be carried out at no cost to integral evaluation.

Second, we also consider two schemes to discard high-angular momentum (HAM) functions from the ABS: in addition to the scheme used by previous automated schemes (refs (14) and (17)), we also discuss a first-principles scheme, which has been previously discussed only in the context of optimized ABSs.^27^ As we demonstrate in this work, the HAM functions can be safely discarded in global fitting methods, as they have a negligible effect on total and relative energies.

The layout of this work is the following. Next, in Section 2, we discuss the theory behind the present approach: the contraction of the ABS is discussed in Section 2.1, while the pruning of HAM functions is discussed in Section 2.2. The computational details of the benchmarking based on HF and MP2 calculations are listed in Section 3. The results of the study are discussed in Section 4, and the study concludes with a brief summary and discussion in Section 5. Atomic units are used throughout, unless specified otherwise.

## 2 Theory

### 2.1 Contracted Sets

For clarity, we reuse the notation of Kállay,^26^ who writes eq 2 in the form
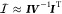
4where  is the ERI tensor, *I*_*μν*,*A*_ = (*μν*|*A*) are the three-index integrals, and *V*_*A*,*B*_ = (*A*|*B*) are the two-index Coulomb overlap integrals. Equation 4 can be expressed in a more compact form in the orthogonalized ABS: defining orthogonalized three-index integrals,
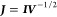
5eq 4 is given simply by
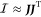
6

Kállay^26^ pointed out that the size of the auxiliary basis can be reduced by a SVD of ***J*** by throwing out vectors with suitably small singular values. The singular values can be computed as the eigenvalues of the symmetric matrix
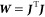
7which has the dimension *N*_aux_ × *N*_aux_, where *N*_aux_ is the number of auxiliary basis functions. Because the number of auxiliary functions typically scales as the number of orbital basis functions, the matrix ***W*** defined by eq 7 can be diagonalized even in large calculations.

In this work, we use the method described above to contract the atomic auxiliary basis. This merely requires forming ***W*** for each element, choosing the contraction based on the singular values, and repeating the procedure for all elements in the basis set.

Because the ***W*** matrix defined by eq 7 is obtained by summing all of the OBS products on the element, ***W*** turns out to have a special angular structure. The only significant values are found when the angular momenta of the bra and ket basis functions coincide, *l*′ = *l* and *m*′ = *m*, with the other elements of ***W*** being numerically zero. As one could expect, all spatial directions are therefore averaged in ***W***.

Because of this special structure, it suffices to compute, e.g., the *m*′ = 0 = *m* subblock of the *l* = *l*′ angular submatrix of ***W***, which we denote as ***L***, which contains information about the importance of the radial auxiliary functions of angular momentum *l*.

The eigendecomposition of ***L*** then yields
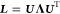
8where **Λ** is a diagonal matrix of eigenvalues, and ***U*** is the corresponding matrix of eigenvectors. Now, we can choose a reduced ABS corresponding to some precision ϵ by including only those columns ***U***_*i*_ that correspond to eigenvalues λ_*i*_ ≥ ϵ. Such an ABS has guaranteed accuracy, as the three-index integrals are reproduced to the precision specified by ϵ.^26^

The contraction algorithm is thus the following:

1. Define the contraction threshold ϵ and read in the OBS and the ABS.
2. Loop over atoms, and the atomic angular momentum *l* in the ABS
  (a) Form the *l* = *l*′ and *m* = 0 = *m*′ angular subblock of ***W*** from eq 7, denoted as ***L***.
  (b) Compute the eigenvectors ***U***_*i*_ with eigenvalues λ_*i*_ of ***L***.
  (c) Include the eigenvectors ***U***_*i*_ in the auxiliary basis if their eigenvalues satisfy λ_*i*_ ≥ ϵ.
  (d) Convert the eigenvectors into the original nonorthonormal basis by ***C*** = ***V***^–1/2^***U***, where ***V***^–1/2^ is the orthogonalizing matrix of eq 5.

To extract the contraction coefficients of a GTO auxiliary basis, a further step is necessary. As Gaussian basis functions are by convention tabulated in terms of primitives normalized in the overlap metric, while the coefficients of the eigenvectors ***C*** formed above correspond to Coulomb normalized primitives, one has to convert the contraction coefficients into the expected normalization. This is achieved by scaling the coefficient of the μth Gaussian primitive in the *i*th auxiliary function, *C*_*μi*_, by the square root of the corresponding Gaussian exponent, , see the Appendix for a derivation.

### 2.2 Pruned Angular Momentum

By default, automatically generated basis sets arising from a large orbital basis set may contain functions of extremely HAM, as an orbital basis with maximum angular momentum *l*_OBS_^max^ will give rise to orbital products with maximum angular momentum 2*l*_OBS_^max^. For instance, a polarized quintuple-ζ basis for the oxygen atom (*l*_OBS_^max^ = 5) will lead to an auxiliary basis set with up to *l* = 10 functions.

Such functions are problematic in Gaussian-basis programs, as most programs do not implement integrals to such HAM. Moreover, since Gaussian integrals are typically computed in the Cartesian Gaussian basis, computing integrals with HAM functions is extremely costly: the number of Cartesian functions of angular momentum *l* is (*l* + 1)(*l* + 2)/2 compared to 2*l* + 1 for spherical functions. Furthermore, the HAM functions’ contributions to total ground state energies tend to be negligible. This motivates removing them from the ABS altogether by a pruning procedure.

The question of what HAM functions can be removed is best approached by asking the opposite question: what are the functions that should never be pruned from the ABS?

Even in highly excited states, most of the total energy arises from orbitals that are occupied in the atomic ground state. The correct description of occupied orbitals being of key importance, the pruned auxiliary basis set should therefore reproduce (i) the highly energetic interactions of the atom’s occupied orbitals with each other, as well as (ii) the interactions of the atom’s occupied orbitals with its unoccupied orbitals, which are important for polarization and correlation effects in polyatomic systems.

Criterion (i) is automatically satisfied if all products of occupied orbitals are correctly reproduced by the auxiliary basis set. Therefore, we demand that the maximum angular momentum of the functions kept in the auxiliary basis, *l*_keep_^max^, is at least two times the maximum angular momentum of occupied shells in the ground state of the atom *l*_occ_^max^, *l*_keep_^max^ ≥ 2*l*_occ_^max^.

Yang et al.^14^ define *l*_occ_^max^ as having the values 0, 1, 2, and 3 for *Z* ≤ 2, 3 ≤ *Z* ≤ 18, 19 ≤ *Z* ≤ 54, and *Z* ≥ 55, respectively, while Stoychev et al.^17^ use the ranges *Z* ≤ 2, 3 ≤ *Z* ≤ 20, 21 ≤ *Z* ≤ 56, and *Z* ≥ 57, respectively. That is, while both Yang et al.^14^ and Stoychev et al.^17^ include *p* functions for the pre-*d* Li and Be atoms, the former likewise add *d* and *f* functions for the pre-*d* and pre-*f* alkali and alkaline atoms, respectively, while the latter do not. We follow Yang et al.^14^ and include *d* and *f* functions for K and Ca, and Cs and Ba, respectively.

Criterion (ii) for the minimal accuracy of the auxiliary basis set is satisfied if the auxiliary basis is able to accurately describe products of an occupied atomic orbital with any other orbital. Denoting the maximum angular momentum of the orbital basis by *l*_OBS_^max^, the second condition is seen to be satisfied by *l*_keep_^max^ ≥ *l*_occ_^max^ + *l*_OBS_^max^.

The two criteria must be satisfied simultaneously, which is why we choose
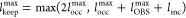
9where *l*_inc_ is an adjustable parameter. As far as we are aware, this criterion has not been studied in the literature in the context of automatic generation of auxiliary basis sets, although a similar discussion can be found in the work of Weigend^27^ discussing the optimization of auxiliary basis sets. (The implementation of the atomic Cholesky procedures of Aquilante et al.^13^ and Aquilante et al.^15^ in the OpenMolcas program^28^ does include optional pruning of the angular momentum to a maximum defined by the auxiliary basis sets of Weigend;^27^ however, this procedure does not appear to be fully documented.)

In contrast, the work of Yang et al.^14^ on Coulomb fitting limited the maximum angular momentum by
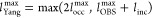
10and employed *l*_inc_ = 1 for all elements, while Stoychev et al.^17^ similarly employed eq 10 with *l*_inc_ = 1 for elements up to Ar and 2 for other elements.

Our truncation eq 9 is seen to have important differences from that used by Yang et al.^14^ and Stoychev et al.^17^ (eq 10): eq 9 includes the atomic angular momentum and therefore naturally employs a larger angular momentum cutoff for heavier elements.

We compare the two schemes for pruning the angular momentum defined by eqs 9 and 10 in Section 4.1, examining various values for *l*_inc_ and comparing the results to ones obtained with the full, unpruned, and uncontracted autogenerated auxiliary basis.

## 3 Computational Details

The computational details of this work are similar to those of ref (18). As in the previous work, we examine the accuracy of the scheme on the triple-ζ to quintuple-ζ nZaPa-NR basis sets of Ranasinghe and Petersson,^29^ for which optimized auxiliary basis sets have not been reported in the literature. These OBSs were obtained from the Basis Set Exchange,^30^ in whose Python backend we previously implemented^18^ the AutoAux procedure of Stoychev et al.,^17^ which is the scheme employed in the Orca program^8^ and which is again used as a state-of-the-art point of reference. In contrast to ref (18), where all ABSs were generated for fully uncontracted OBSs, the ABSs used in this work are generated for contracted OBSs.

The full primitive ABSs were generated with ERKALE^31,32^ following the procedure of ref (18), employing the threshold τ = 10^–7^ for the pivoted Cholesky decomposition. Unless specified otherwise, the orbital products were prescreened with a pivoted Cholesky decomposition of the ERI tensor, as recommended in ref (18).

The contraction and reduction schemes presented in Section 2 were implemented in ERKALE^31,32^ in this work, and they are freely and openly available on GitHub. The employed values for the contraction and reduction parameters, ϵ and *l*_inc_, respectively, are discussed in Section 4.

Our previous work^18^ studied HF and MP2 total energies in the nonmultireference part of the W4–17 test set^33^ and showed that their RI/DF errors can be made negligible by the use of suitably large primitive ABSs. As the ABSs generated by the previously suggested automated procedure are already known to be transferable,^18^ and the contraction and reduction schemes of Section 2 are based on mathematical principles, we study here the chemically diverse subset of first- and second-row molecules from the database of Weigend and Ahlrichs,^34^ which has been previously used to assess DF/RI auxiliary basis sets for Hartree–Fock.^27^ We limit the calculations to molecules containing at most nine atoms, which suffice to demonstrate the accuracy of the reduced basis sets.

As the employed spin states for the molecules are not documented in refs (27) and (34), we decided to run HF and MP2 calculations for the Al (doublet), Be, B (doublet), Cl (doublet), C (triplet), F (doublet), H (doublet), Li (doublet), Mg, Na (doublet), N (quartet), O (triplet), P (quartet), Si (triplet), and S (triplet) atoms and the AlN (triplet), BeS (triplet), Cl_2_, ClF, CO, F_2_, H_2_, HCl, HF, Li_2_, LiCl, LiF, LiH, MgF (doublet), N_2_, NaCl, NaF, NaH, P_2_, S_2_ (triplet), BeH_2_, CO_2_, CS_2_, H_2_O, HCN, HCP, HNC, HNO, HSH, Li_2_O, LiSLi, MgCl_2_, MgF_2_, MgH_2_, Na_2_O (triplet), Na_2_S, OF_2_, SF_2_, SiO_2_, SiS_2_, AlCl_3_, AlF_3_, AlH_3_, Be_4_, BF_3_, BH_3_, C_2_H_2_, CH_2_O, ClF_3_, H_2_O_2_, HNO_2_, HSSH, Mg_4_, N_2_H_2_, N_4_, Na_3_N (triplet), Na_3_P (triplet), NF_3_, NH_3_, PF_3_, PH_3_, PLi_3_, Al_2_O_3_, Al_2_S_3_, CF_4_, CH_2_O_2_, CH_3_N, CH_4_, HNO_3_, S_5_, SF_4_, SiCl_4_, SiF_4_, SiH_4_, Be_2_F_4_, Be_2_H_4_, BH_3_CO, C_2_H_3_N, C_2_H_4_, CH_3_OH, H_2_CO_3_, LiBH_4_, N_2_H_4_, NH_4_F, PF_5_, H_2_SO_4_, SF_6_, B_2_H_6_, B_4_H_4_, BH_3_NH_3_, C_2_H_6_, C_4_H_4_, H_3_PO_4_, Li_4_Cl_4_, Li_4_H_4_, Li_8_, BeC_2_H_6_, and BeF_2_O_2_H_4_ molecules. Altogether, the assessment therefore includes 113 systems composed of 15 atoms and 98 molecules.

Conventional HF and MP2 calculations were performed for these molecules with the Gaussian’16 program;^35^ modifications of the basis set were disabled with IOp(3/60 = −1). The RI/DF calculations were carried out with Psi4.^7^ The basis set linear dependence threshold was set to 10^–7^ in both programs.

As our test systems only include first- and second-row molecules, the same value of *l*_inc_ is used for all elements in eq 10 in our test calculations; note that Yang et al.^14^ and Stoychev et al.^17^ similarly use *l*_inc_ = 1 for all elements up to argon.

## 4 Results

### 4.1 Detailed Analysis in the 3ZaPa-NR Basis Set

We begin by analyzing how RI/DF errors in HF and MP2 total and atomization energies are affected by contracting the auxiliary basis set according to the procedure of Section 2.1, when the 3ZaPa-NR OBS is used. Because the DF/RI error is an extensive quantity, we examine errors in total energies *per electron* and errors in atomization energies *per atom.*

**Figure 1:**
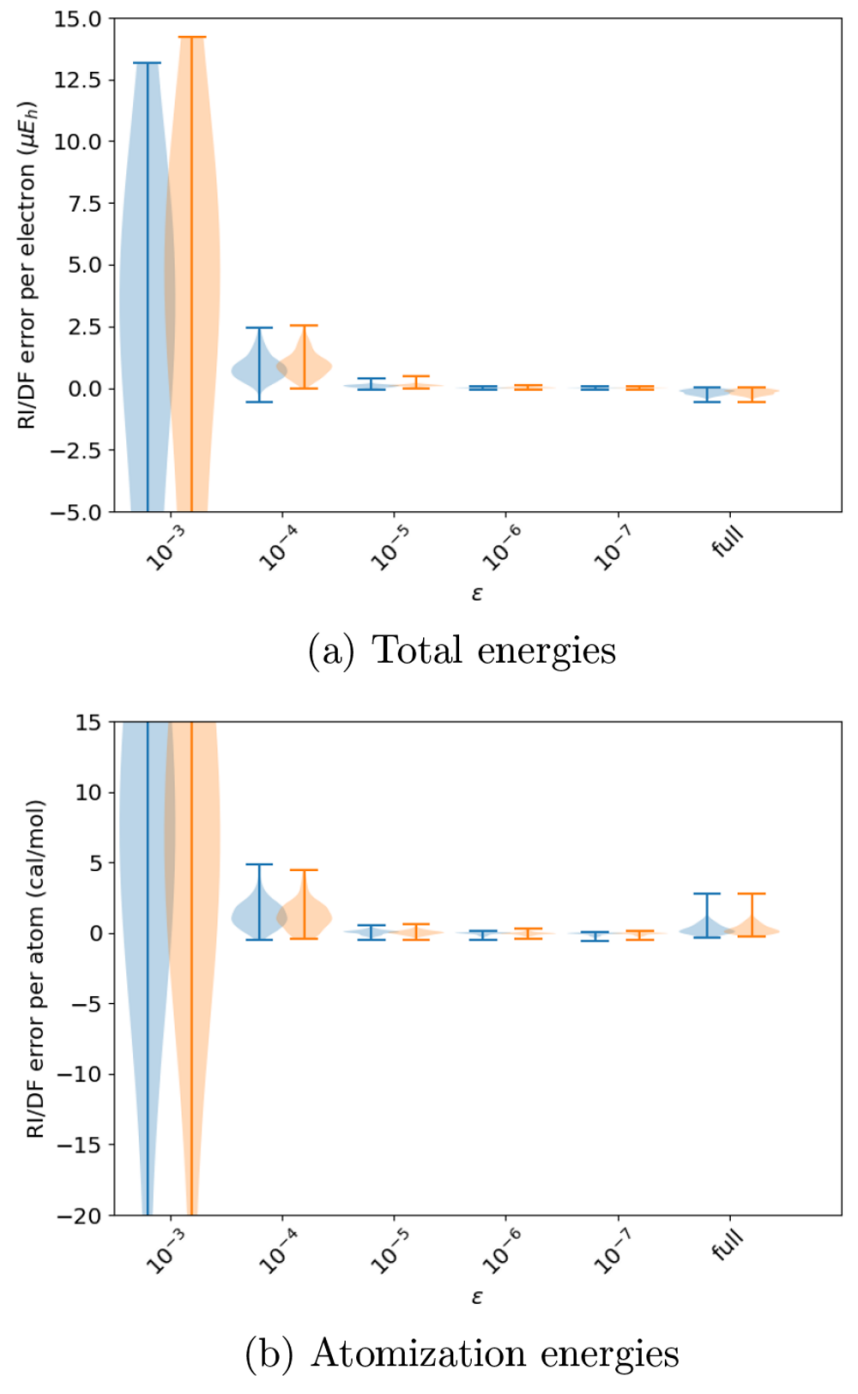
Effect of contraction on the RI/DF errors in the total HF (cyan) and MP2 (orange) and atomization energies of the studied systems in the 3ZaPa-NR basis set. The last entry in the violin plot shows the error distribution for the full, unpruned, and uncontracted parent auxiliary basis set.

The RI/DF errors of the studied test set are shown as a function of the contraction threshold in Figure 1 for the HF and MP2 total and atomization energies. The RI/DF errors converge rapidly when the contraction threshold ϵ is decreased. We observe that already the contraction threshold ϵ = 10^–4^ affords RI/DF errors in the order of a few *μE*_*h*_ per electron in total energies and a few cal/mol per atom in atomization energies, which are negligible compared to the truncation error of the OBS and the inherent errors in the employed levels of theory.

It is also important to note here that the HF and MP2 errors behave very similarly as a function of ϵ. This result is fully expected for the contraction based on an SVD of the three-index integrals: because all integral classes are treated on the same footing, the error is not expected to depend on the examined level of theory.

Interestingly, the RI/DF errors afforded by small values of ϵ are smaller than those obtained with the full parent auxiliary basis set. This difference can likely be attributed to the better conditioning of the contracted auxiliary basis set. Unlike the parent ABS, the functions of the contracted ABS are orthonormal on each atom, leading to improved numerical stability in the RI/DF method when the contracted ABS is used in polyatomic calculations; this in turn reduces numerical errors. In addition, the contracted ABS also contains fewer functions, which likewise improves the numerical stability.

**Figure 2:**
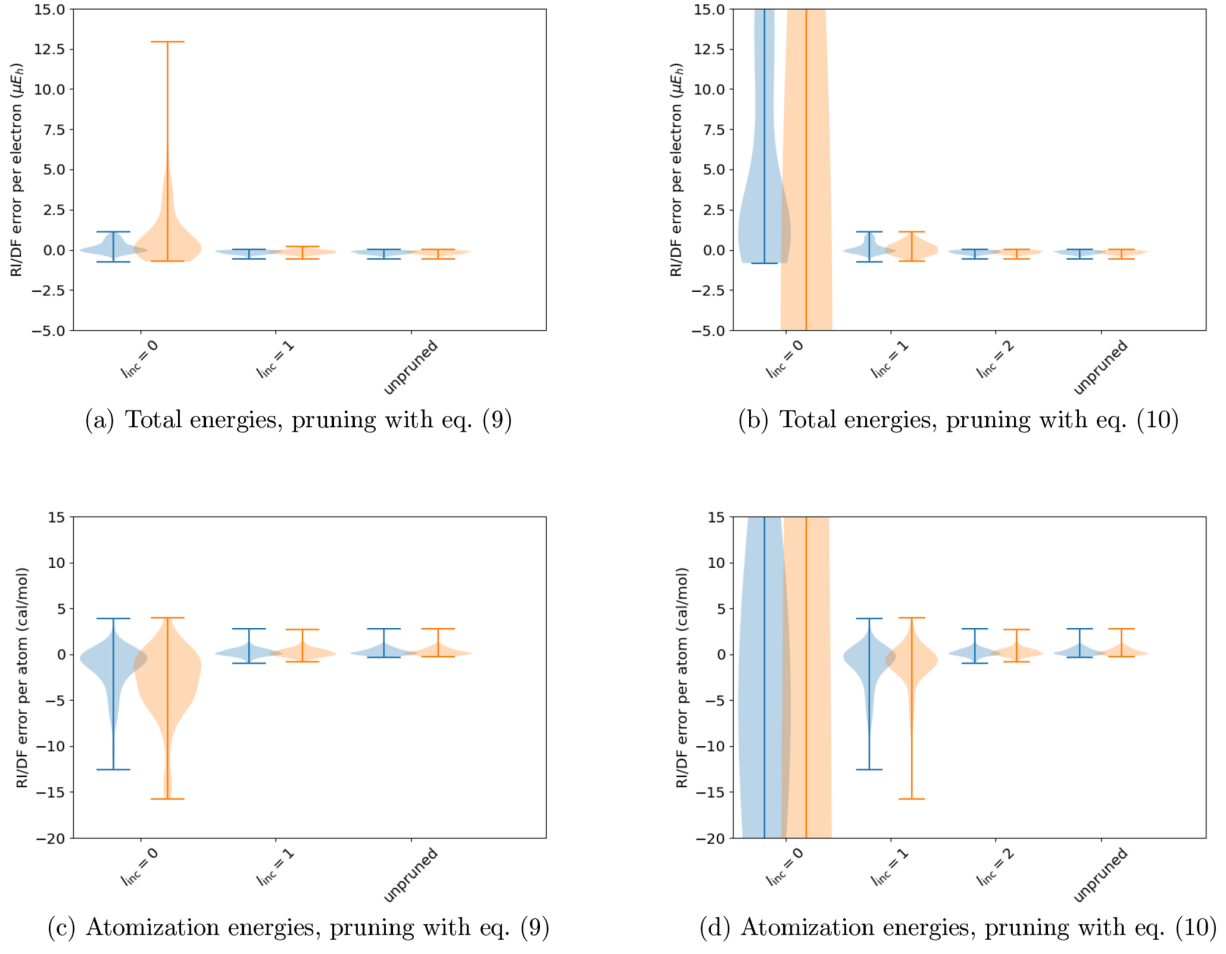
Effect of pruning high-angular-momentum functions according to eqs 9 and 10 on the RI/DF errors HF (cyan) and MP2 (orange) total and atomization energies of the studied systems in the 3ZaPa-NR basis set. The last entries in the violin plots show the error distribution for the full, unpruned, and uncontracted parent auxiliary basis set.

We move on to studying the effect of pruning HAM auxiliary functions according to eqs 9 and 10. The results of these procedures on the HF and MP2 total and atomization energies are shown in Figure 2.

The value *l*_inc_ = 0 yields good results with eq 9: although the MP2 total energy exhibits a small maximum absolute error of some 13 *μE*_*h*_ per electron (which arises for H_2_), the errors in HF total energies appear to be small and close to those of the full original ABS.

In contrast, the value *l*_inc_ = 0 yields unacceptably large errors for eq 10: for instance, the MP2 total energy of the nitrogen atom is off by −223 *μE*_*h*_ per electron, and the MP2 atomization energy of P_2_ is off by −893 cal/mol per atom.

Because eq 10 clearly exhibits the wrong physical behavior, for the rest of this work, we consider only eq 9 for pruning the HAM functions. With this scheme, *l*_inc_ = 0 already yields good accuracy, and the values with *l*_inc_ = 1 are almost converged to the full uncontracted and unpruned ABS, which is reproduced by *l*_inc_ = 2 for the presently considered triple-ζ 3ZaPa-NR basis set.

### 4.2 Performance in Larger Basis Sets

We observe from the analysis of Section 4.1 that contraction thresholds ϵ ≲ 10^–4^ afford suitably accurate total and atomization energies, while pruning HAM functions with eq 9 also works well. In this section, we examine the accuracy of the compound procedure, where the ABS is both contracted and pruned of HAM functions.

**Figure 3:**
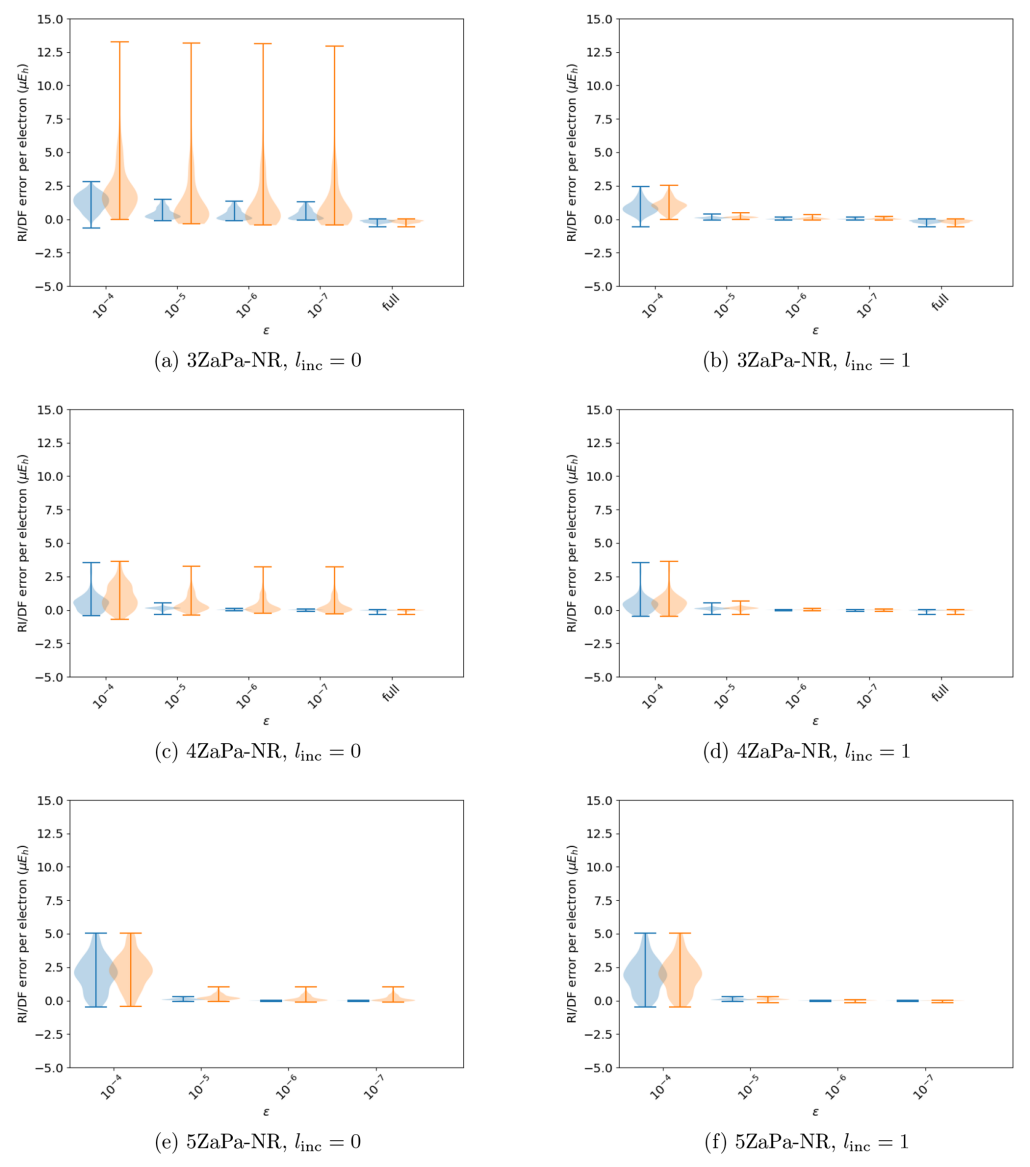
Effect of contraction on the RI/DF errors in the HF (cyan) and MP2 (orange) total energies of the studied systems in various basis sets, when the auxiliary basis is also pruned with eq 9 with *l*_inc_ = 0 or *l*_inc_ = 1. The last entry in the 3ZaPa-NR and 4ZaPa-NR plots show the error distributions for the full, unpruned and uncontracted parent auxiliary basis set.

Starting with the total energies shown in Figure 3, we observe for all of the OBSs that the ABS contracted and pruned with eq 9 with *l*_inc_ = 1 affords negligible errors: as long as the contraction threshold ϵ ≲ 10^–5^, the errors in the HF and MP2 total energies are of the order of 1 *μE*_*h*_ per electron or smaller.

We also observe that for a fixed value of *l*_inc_, the maximum RI/DF errors go down when the size of the OBS is increased. While *l*_inc_ = 0 leads to errors in the total energy of the order of 13 *μE*_*h*_ per electron with the triple-ζ 3ZaPa-NR OBS, the error decreases to  per electron in the quadruple-ζ 4ZaPa-NR OBS, and  per electron in the quintuple-ζ 5ZaPa-NR OBS.

Given that the increase in the size of the OBS is accompanied by an increase in the maximum angular momentum of the corresponding ABS (eq 9), a likely explanation for the decreasing error with increasing OBS is that the one-center approximation that is the cornerstone of the DF/RI method^12^ is becoming more accurate: energetically important two-center products of OBS functions are captured to better and better accuracy, when the ABS becomes larger and larger. Increasing the cardinal number of the OBS adds higher polarization shells in the autogenerated ABS and also adds more radial functions to the lower angular momenta in the ABS.

**Figure 4:**
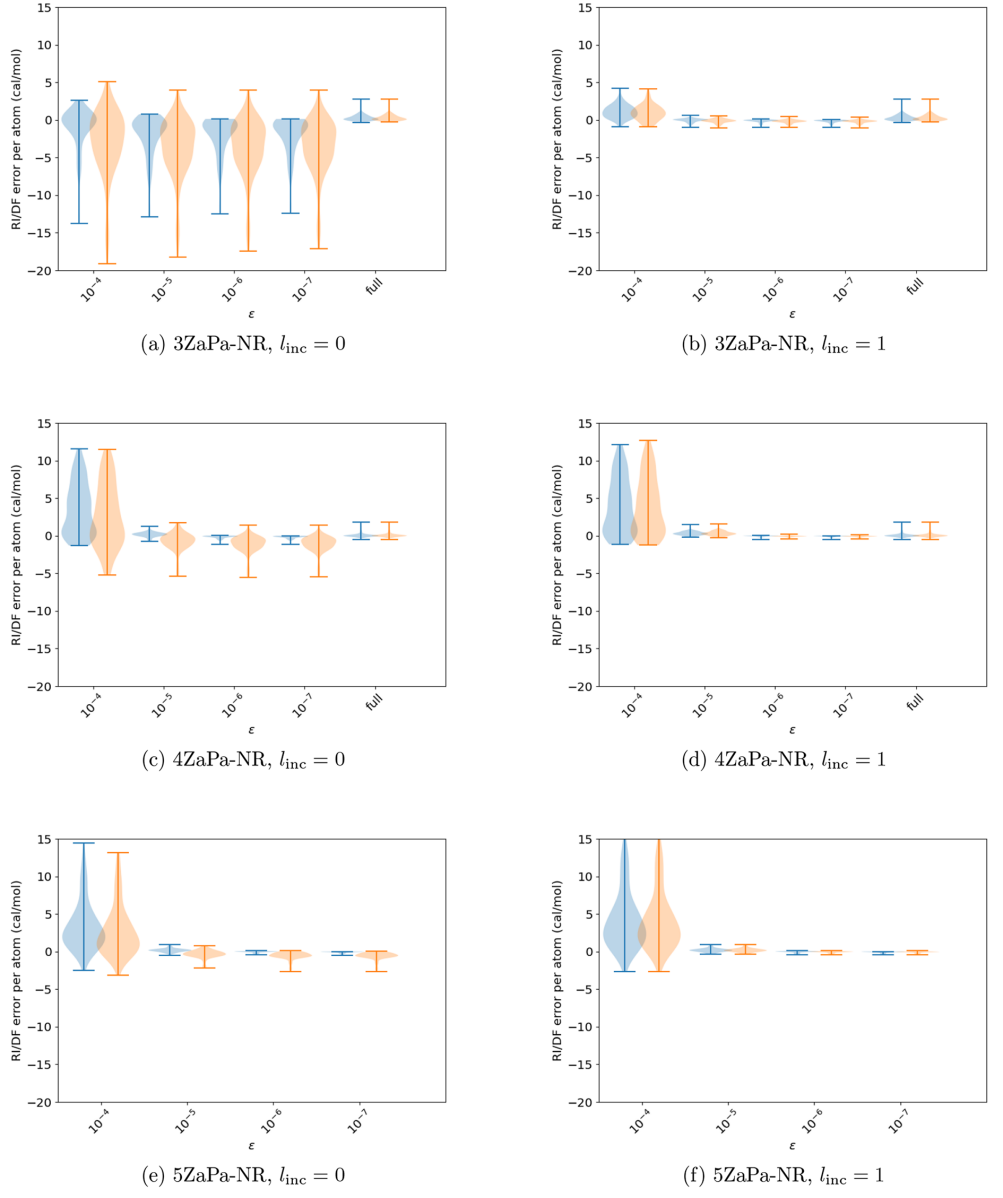
Effect of contraction on the RI/DF errors in the HF (cyan) and MP2 (orange) atomization energies of the studied molecules in various basis sets, when the auxiliary basis is also pruned with eq 9 with *l*_inc_ = 0 or *l*_inc_ = 1. The last entry in the 3ZaPa-NR and 4ZaPa-NR plots shows the error distributions for the full, unpruned, and uncontracted parent auxiliary basis set.

The corresponding results for atomization energies are shown in Figure 4. The errors in atomization energies of the order of cal/mol per atom are orders of magnitude smaller than the usual limit of chemical accuracy of 1 kcal/mol. We again note the increasing accuracy of the ABS with fixed *l*_inc_ in increasing size of the OBS, which is clear in the small-ϵ data for *l*_inc_ = 0. Excellent accuracy is achieved with *l*_inc_ = 1 and ϵ ≲ 10^–5^ in all OBSs.

### 4.3 Size Reductions

Having established that an excellent level of accuracy can be reached with the DF/RI approximation with the presently considered pruning and contraction technique for the automatically generated ABSs, we can proceed to discussing the associated reductions in the size of the ABS. As was already mentioned above, the computational cost of the DF/RI technique is linear in the size of the ABS; therefore, discussion on the cost of the DF/RI technique usually boils down to the examination of the ratio
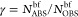
11that is, the number of auxiliary functions divided by the number of orbital functions.

In the following, we examine the accuracy and cost of automatically generated ABSs with the present method, considering simultaneous contraction with ϵ ∈ [10^–4^, 10^–5^, 10^–6^] and pruning with *l*_inc_ ∈ [0, 1], which yielded the good results in Section 4.2. We compare the results to those of the AutoAux procedure of Stoychev et al.^17^

**Figure 5:**
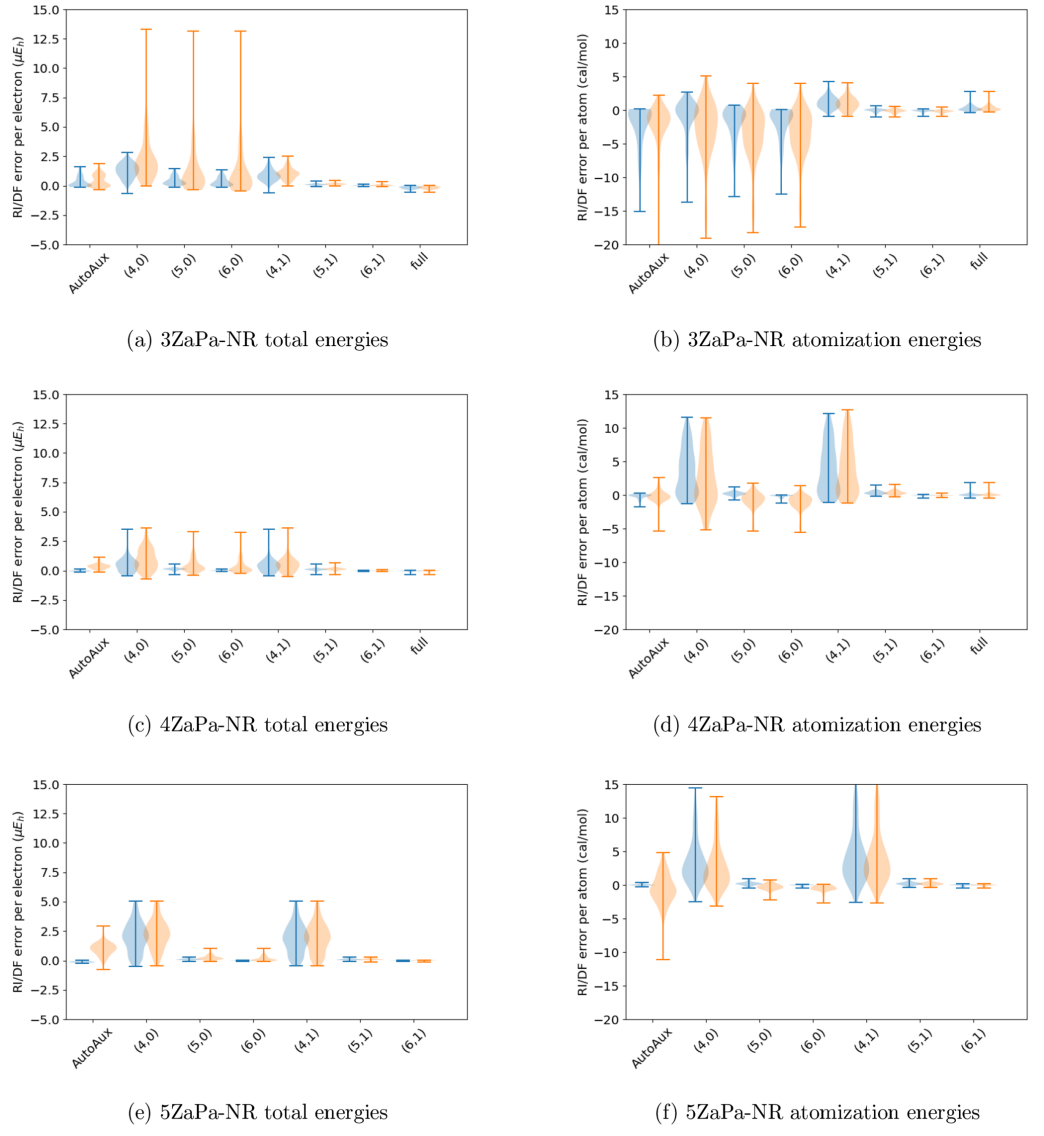
Summary of the performance of the various automated schemes for automated auxiliary basis set construction across various orbital basis sets: the AutoAux procedure of Stoychev et al.,^17^ as well as contracted and pruned basis sets with the parameters ϵ = 10^–*x*^ and *l*_inc_ = *y* specified with the shorthand (*x*, *y*). The last entry in the 3ZaPa-NR and 4ZaPa-NR plots show the error distributions for the full, unpruned, and uncontracted parent auxiliary basis set.

The accuracy of these ABSs is shown in Figure 5. From these data, we can identify reasonable combinations of the truncation parameters that appear to be balanced in the contraction and pruning of HAM functions. The choice ϵ = 10^–4^ and *l*_inc_ = 0 yields a “small” ABS, while ϵ = 10^–5^ and *l*_inc_ = 1 leads to a “large” ABS, and ϵ = 10^–6^ and *l*_inc_ = 1 yields a “verylarge” ABS.

The AutoAux basis is much more accurate for HF than for MP2 in large OBSs, and the method does not offer a straightforward way to improve the accuracy of the generated ABS. In contrast, the “small”, “large”, and “verylarge” ABSs obtained with the above procedure provide a sequence of improving accuracy in all studied OBSs. With the exclusion of the “small” ABS in the 3ZaPa-NR OBS, they also afford similar accuracy at both the HF and MP2 levels of theory.

**Figure 6:**
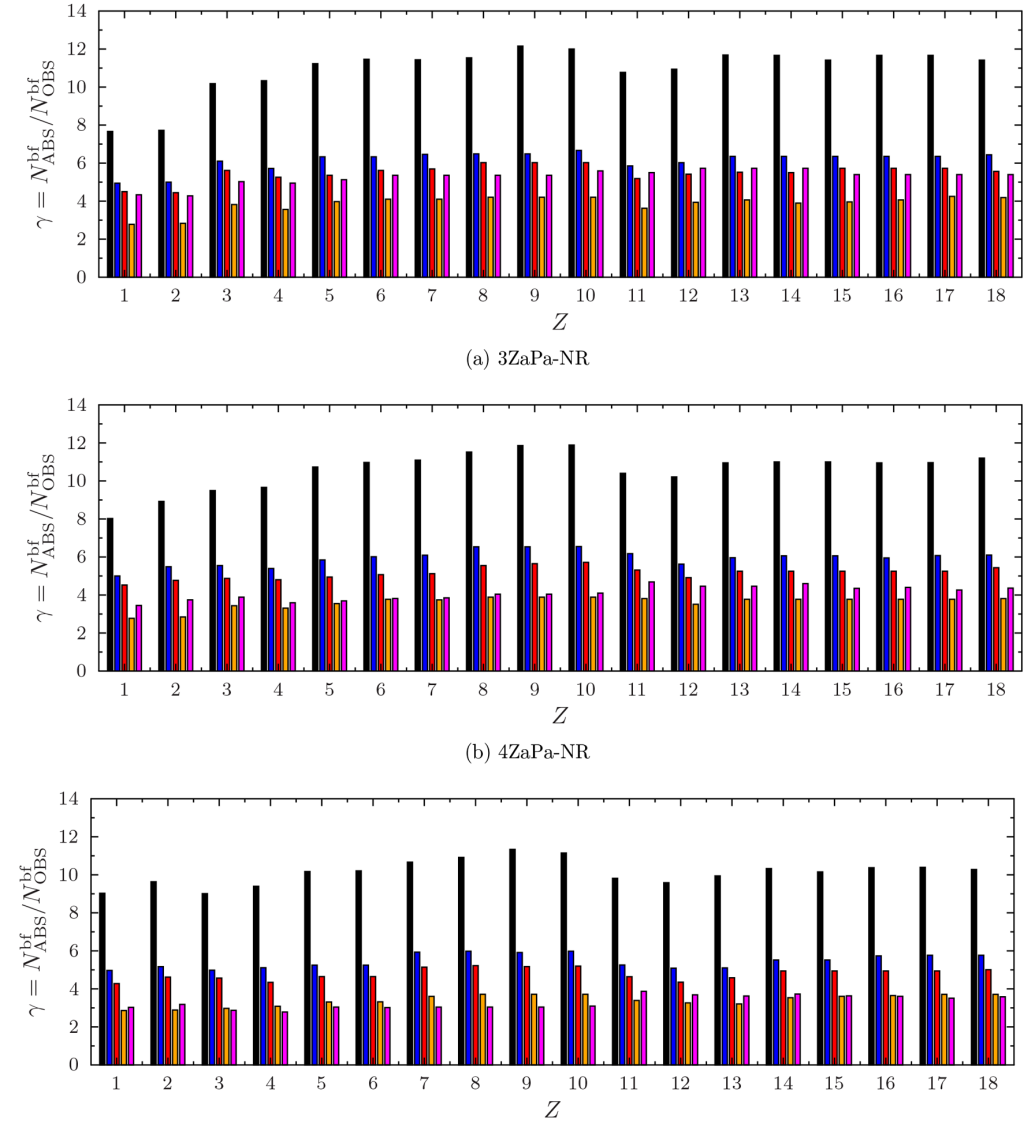
Sizes of autogenerated auxiliary basis for 3ZaPa-NR, 4ZaPa-NR, and 5ZaPa-NR for H–Ar. Reading in order from the left, the original primitive auxiliary basis set, generated by the procedure of ref (18), is shown in black. The verylarge (ϵ = 10^–6^ and *l*_inc_ = 1), large (ϵ = 10^–5^ and *l*_inc_ = 1), and small (ϵ = 10^–4^ and *l*_inc_ = 0) auxiliary sets are shown in blue, red, and orange, respectively. For comparison, the size of the ABS generated with the AutoAux procedure of Stoychev et al.^17^ is shown in magenta.

The ratios γ defined by eq 11 for the various ABSs are shown in Figure 6, where the size of the full, unpruned, and uncontracted autogenerated ABS is also shown for reference. The minimum and maximum values of γ are also given in Table 1 for all the corresponding ABSs.

**Table 1:** Ranges of γ Defined by eq 11 of Various Automatically Generated ABSs

	full		“verylarge”		“large”		“small”		AutoAux	
	min γ	max γ	min γ	max γ	min γ	max γ	min γ	max γ	min γ	max γ
3ZaPa-NR	7.7	12.2	4.9	6.7	4.4	6.0	2.8	4.2	4.3	5.7
4ZaPa-NR	8.0	11.9	5.0	6.5	4.5	5.7	2.8	3.9	3.5	4.7
5ZaPa-NR	9.0	11.3	5.0	6.0	4.3	5.2	2.9	3.7	2.8	3.9

As was already discussed in ref (18), the full autogenerated auxiliary basis sets are large: we see from Figure 6 and Table 1 that the ratio γ varies from 8 to 12 in the full ABS. However, we also see that the contraction and pruning procedure of this work leads to significant reductions in the size of the ABSs.

Already going from the full ABS to the “verylarge” ABS is seen to roughly halve the number of auxiliary functions, indicating potential for significant savings with negligible errors, as shown by the related accuracy data in Figure 5.

A further reduction is achieved by going to the “large” ABS, which still affords consistently small errors (Figure 5). The “small” ABS, in turn, exhibits γ in the range 3–4 for all of the OBSs, enabling quick calculations with somewhat larger errors, which may still be sufficiently small for practical applications.

### 4.4 Role of the Primitive Auxiliary Basis

Having demonstrated the accuracy of the reduction scheme, we examine the role of the primitive auxiliary basis fed into the contraction procedure. We compare contracted ABSs formed with and without the prescreening of the orbital products, which was performed for the results above with a pivoted Cholesky decomposition of the ERI tensor as recommended in ref (18).

Comparing the compositions of the contracted ABSs for the 3ZaPa-NR and 4ZaPa-NR OBSs shown in Table 2, we observe that despite the large differences in the number of Gaussian primitives in the ABSs generated with and without the orbital product screening—which were already observed in ref (18)—in both cases the contracted ABSs turn out to have the same composition, that is, the same number of contracted functions.

**Table 2:** Compositions of Contracted ABSs for 3ZaPa-NR and 4ZaPa-NR OBSs with ϵ = 10^–5,^^a^

(a) 3ZaPa-NR				
	without prescreening		with prescreening	
	primitive	contracted	primitive	contracted
H	15s14p10d4f1g	9s7p6d3f1g	15s11p10d4f1g	9s7p6d3f1g
He	17s16p11d4f1g	8s7p6d3f1g	16s12p9d4f1g	8s7p6d3f1g
Li	24s23p20d17f9g4h1i	11s9p9d7f6g3h1i	22s19p16d12f9g4h1i	11s9p9d7f6g3h1i
Be	24s23p20d18f9g4h1i	11s9p8d7f5g3h1i	24s20p15d13f9g4h1i	11s9p8d7f5g3h1i
B	24s25p22d20f10g4h1i	10s9p9d7f5g3h1i	23s21p18d15f10g4h1i	10s9p9d7f5g3h1i
C	26s25p23d20f10g4h1i	11s9p9d7f6g3h1i	23s21p18d14f10g4h1i	11s9p9d7f6g3h1i
N	25s25p23d21f10g4h1i	11s10p9d7f6g3h1i	24s23p20d14f9g4h1i	11s10p9d7f6g3h1i
O	25s26p23d21f11g4h1i	12s10p10d8f6g3h1i	25s23p20d15f9g4h1i	12s10p10d8f6g3h1i
F	26s26p23d23f11g4h1i	12s10p10d8f6g3h1i	25s23p21d16f10g4h1i	12s10p10d8f6g3h1i
Ne	26s26p24d22f11g4h1i	12s10p10d8f6g3h1i	25s24p22d16f10g4h1i	12s10p10d8f6g3h1i
Na	31s29p28d26f15g5h1i	13s10p10d7f7g4h1i	29s27p23d15f11g5h1i	13s10p10d7f7g4h1i
Mg	30s32p29d28f15g5h1i	14s11p10d8f7g4h1i	30s28p24d16f11g5h1i	14s11p10d8f7g4h1i
Al	30s32p31d29f16g5h1i	14s11p11d8f7g4h1i	30s30p26d18f11g5h1i	14s11p11d8f7g4h1i
Si	31s31p29d29f16g5h1i	14s11p11d8f7g4h1i	30s29p25d18f12g5h1i	14s11p11d8f7g4h1i
P	31s31p30d28f16g5h1i	14s12p11d9f7g4h1i	30s29p25d18f12g5h1i	14s12p11d9f7g4h1i
S	30s31p29d28f16g5h1i	14s12p11d9f7g4h1i	30s30p26d18f12g5h1i	14s12p11d9f7g4h1i
Cl	30s31p29d28f16g5h1i	14s12p11d9f7g4h1i	30s29p26d19f12g5h1i	14s12p11d9f7g4h1i
Ar	30s31p29d28f15g5h1i	13s12p11d8f7g4h1i	29s30p25d18f12g5h1i	13s12p11d8f7g4h1i

(b) 4ZaPa-NR				
	without prescreening		with prescreening	
	primitive	contracted	primitive	contracted
H	18s16p15d12f8g4h1i	11s9p8d7f6g3h1i	16s13p13d11f7g4h1i	11s9p8d7f6g3h1i
He	20s19p18d14f8g4h1i	10s9p9d7f6g3h1i	20s15p13d12f8g4h1i	11s9p9d7f6g3h1i
Li	26s25p23d22f19g11h7i4j1k	13s11p10d8f7g6h5i3j1k	25s21p17d14f13g10h7i4j1k	13s11p10d8f7g6h5i3j1k
Be	26s25p22d23f21g11h7i4j1k	11s10p10d8f7g6h5i3j1k	26s21p17d14f12g10h7i4j1k	11s10p10d8f7g6h5i3j1k
B	27s27p24d24f22g12h8i4j1k	11s11p10d9f7g6h5i3j1k	26s23p19d17f15g11h8i4j1k	11s11p10d9f7g6h5i3j1k
C	28s27p25d24f23g13h8i4j1k	11s11p10d9f8g6h5i3j1k	28s23p20d17f15g11h8i4j1k	11s11p10d9f8g6h5i3j1k
N	29s28p27d25f25g14h8i4j1k	13s11p12d10f8g6h5i3j1k	28s26p23d18f15g12h8i4j1k	13s11p12d10f8g6h5i3j1k
O	28s28p26d25f25g14h9i4j1k	13s12p11d9f8g7h6i3j1k	28s25p22d18f15g12h9i4j1k	13s12p11d9f8g7h6i3j1k
F	28s29p27d26f25g15h9i4j1k	13s12p11d10f8g7h6i3j1k	27s27p23d19f16g12h8i4j1k	13s12p11d10f8g7h6i3j1k
Ne	29s29p27d26f25g15h8i4j1k	13s12p12d10f8g7h6i3j1k	28s27p24d19f16g12h8i4j1k	13s12p11d10f8g7h6i3j1k
Na	33s34p31d31f31g17h8i4j1k	16s13p13d10f9g7h6i3j1k	32s32p27d18f13g12h8i4j1k	15s13p13d10f9g7h6i3j1k
Mg	32s33p30d30f30g16h8i4j1k	15s13p12d9f8g6h6i3j1k	31s31p26d18f13g11h8i4j1k	15s13p12d9f8g6h6i3j1k
Al	33s33p33d32f31g18h8i4j1k	15s13p12d10f9g7h6i3j1k	32s32p27d19f14g12h8i4j1k	15s13p12d10f9g7h6i3j1k
Si	32s33p32d32f32g18h8i4j1k	15s13p12d10f9g7h6i3j1k	31s31p27d20f16g12h8i4j1k	15s13p12d10f9g7h6i3j1k
P	32s34p31d31f32g18h8i4j1k	15s13p12d10f9g7h6i3j1k	31s31p27d20f15g12h8i4j1k	15s13p12d10f9g7h6i3j1k
S	32s33p31d31f32g18h8i4j1k	15s13p12d10f9g7h6i3j1k	31s31p27d19f15g13h8i4j1k	15s13p12d10f9g7h6i3j1k
Cl	32s33p31d31f32g18h8i4j1k	15s13p12d10f9g7h6i3j1k	32s31p27d20f15g13h8i4j1k	15s13p12d10f9g7h6i3j1k
Ar	32s33p31d31f31g18h8i4j1k	15s14p13d11f9g7h6i3j1k	31s31p27d19f15g12h8i4j1k	15s14p13d11f9g7h6i3j1k

a The labeling of the angular momentum follows the Gaussian’94 convention, which does not skip the letter *J* for *l* = 7 unlike most other conventions.

However, the use of the prescreening technique is still highly attractive for GTO calculations. Because the integrals are evaluated in terms of the primitive GTOs, reducing the number of primitive functions is key to making the approach computationally efficient.

But, when the present contraction scheme is used with NAOs, it appears that prescreening the orbital products via a pivoted Cholesky decomposition of the ERI tensor is indeed unnecessary: the simpler scheme of simply forming all radial orbital products, choosing the auxiliary basis set that reproduces all of them and then contracting it appears to lead to a similarly compact ABS, which thereby has the same computational cost. The key difference to GTOs is that NAO integrals are evaluated by quadrature, and the contraction can be carried out as a transformation of the NAOs’ expansion coefficients; see ref (22) for details.

## 5 Summary and Discussion

We described an automated scheme for contracting auxiliary basis sets (ABSs) for a given orbital basis set (OBS). The scheme works with any type of atomic basis function: in addition to the Gaussian-type orbitals (GTOs) considered in this work, the scheme can also be straightforwardly applied to other types of atomic basis functions, such as Slater-type orbitals (STOs) and numerical atomic orbitals (NAOs). Our procedure starts from a “full” ABS generated using the algorithm of ref (18). This ABS is then contracted and pruned of high-angular momentum (HAM) functions to produce computationally efficient ABSs.

By default, the scheme of ref (18) employs a pivoted Cholesky decomposition of the electron repulsion integral (ERI) tensor to choose the subset of orbital products from which the auxiliary basis functions are chosen with another pivoted Cholesky decomposition. We found in ref (18) that prescreening the orbital products with a pivoted Cholesky of the ERIs significantly decreases the number of primitive GTOs in the resulting autogenerated auxiliary basis set, leading to better computational efficiency while maintaining the same level of accuracy.

Interestingly, we find that the contracted auxiliary basis set obtained with the singular value decomposition (SVD) of eq 7 comes out similar even if the initial pivoted Cholesky decomposition of the ERIs is not performed, that is, when the full set of orbital products is employed to generate the auxiliary functions. The present scheme thus appears to reproduce a similar number of contracted auxiliary functions regardless of whether any initial screening of orbital products is done.

Although GTOs incur additional cost from contractions, as their integrals are usually evaluated in terms of Gaussian primitives, if NAOs are used, then the present scheme becomes especially interesting. As NAO integrals are evaluated by quadrature, the linear transformation of the input auxiliary functions can be simply carried out to their expansion coefficients; initialization with the pivoted Cholesky of the ERIs to choose the product functions is therefore not needed in the context of NAO calculations. We therefore believe that in addition to the application of the present method to GTO basis sets presented in this work, our scheme will be found to be extremely useful for NAO-based calculations as well. We have recently developed modern software for atomic electronic structure calculations employing high-order numerical methods that allow rapid convergence to the complete basis set (CBS) limit with few numerical basis functions,^21,22,36−38^ and hope to apply these techniques to NAO-based calculations in the near future, where the schemes of ref (18) and this work will be of critical importance.

## Appendix: Conversion of Contraction Coefficients

Let ϕ_*A*_(***r***) denote an unnormalized Gaussian primitive. From the eigendecomposition, one obtains eigenvectors in terms of Coulomb normalized primitives ∑*_A_C*_*A*_^C^*N*_*A*_^C^ϕ_*A*_(***r***), while basis set input is handled in typical programs in terms of overlap normalized primitives ∑*_A_C*_*A*_^o^*N*_*A*_^o^ϕ_*A*_(***r***). Denoting the two normalization coefficients as *N*_*A*_^C^ = (*A*|*A*)^−1/2^ and *N*_*A*_^o^ = ⟨*A*|*A*⟩^–1/2^, where the overlap of two unnormalized Gaussian primitives is^39^

12

and the Coulomb overlap is given by^18^

13

one finds the ratio

14

from which the coefficient follows as

15

that is, the coefficients are multiplied by the square root of the corresponding exponent.
